# Metabolic Profiling as a Screening Tool for Cytotoxic Compounds: Identification of 3-Alkyl Pyridine Alkaloids from Sponges Collected at a Shallow Water Hydrothermal Vent Site North of Iceland

**DOI:** 10.3390/md15020052

**Published:** 2017-02-22

**Authors:** Eydis Einarsdottir, Manuela Magnusdottir, Giuseppe Astarita, Matthias Köck, Helga M. Ögmundsdottir, Margret Thorsteinsdottir, Hans Tore Rapp, Sesselja Omarsdottir, Giuseppe Paglia

**Affiliations:** 1 Faculty of Pharmaceutical Sciences, University of Iceland, Reykjavík 107, Iceland; eydisei@hi.is (E.E.); margreth@hi.is (M.T.); sesselo@hi.is (S.O.); 2 Center for Systems Biology, University of Iceland, Reykjavík 101, Iceland; mam5@hi.is; 3 Denali Therapeutics, South San Francisco, CA 94080, USA; gastarita@gmail.com; 4 Department of Biochemistry and Molecular & Cellular Biology, Georgetown University, Washington, DC 20057, USA; 5 Helmholtz Center for Polar and Marine Research, Alfred-Wegener-Institute, Bremerhaven D-27570, Germany; mkoeck@awi.de; 6 Faculty of Medicine, University of Iceland, Reykjavik 101, Iceland; helgaogm@hi.is; 7 Department of Biology and KG Jebsen Centre for Deep Sea Research, University of Bergen, Bergen 5020, Norway; hans.rapp@bio.uib.no; 8 Center for Biomedicine, European Academy of Bolzano/Bozen, Bolzano 39100, Italy

**Keywords:** marine natural products, sponges, metabolomics, ion mobility, *Haliclona rosea*

## Abstract

Twenty-eight sponge specimens were collected at a shallow water hydrothermal vent site north of Iceland. Extracts were prepared and tested in vitro for cytotoxic activity, and eight of them were shown to be cytotoxic. A mass spectrometry (MS)-based metabolomics approach was used to determine the chemical composition of the extracts. This analysis highlighted clear differences in the metabolomes of three sponge specimens, and all of them were identified as *Haliclona (Rhizoniera) rosea* (Bowerbank, 1866). Therefore, these specimens were selected for further investigation. *Haliclona rosea* metabolomes contained a class of potential key compounds, the 3-alkyl pyridine alkaloids (3-APA) responsible for the cytotoxic activity of the fractions. Several 3-APA compounds were tentatively identified including haliclamines, cyclostellettamines, viscosalines and viscosamines. Among these compounds, cyclostellettamine P was tentatively identified for the first time by using ion mobility MS in time-aligned parallel (TAP) fragmentation mode. In this work, we show the potential of applying metabolomics strategies and in particular the utility of coupling ion mobility with MS for the molecular characterization of sponge specimens.

## 1. Introduction

The ocean covers more than seventy percent of Earth’s surface and harbors enormous biodiversity, largely undiscovered. Iceland is a volcanic island in the North Atlantic Ocean and it is the largest part of the Mid-Atlantic Ridge (MAR) that emerges from the sea [1]. Around the island there are several submarine geothermal active sites hosting a highly diverse fauna. Until now there have been no comprehensive studies on the diversity and bioactivity of marine natural products associated with the organisms found at these sites [2]. Hydrothermal vent fields are known to be found around oceanic ridges worldwide, mainly at great depths. Iceland has a unique vent field site positioned in shallow water, composed of cones and ridges built up for thousands of years by the precipitation of SiO_2_. Hot alkaline fresh water (pH 10 and 72 °C) circulates in this hydrothermal vent site. Furthermore, it is very different from the deeper water vent fields worldwide, both chemically and biologically. These unique vent fields are the very few in the world that are easily accessed by SCUBA diving [3,4]. Sponges (Porifera) are sessile organisms, known to produce bioactive secondary metabolites for their protection as well as for reproduction and communication [5,6,7]. In the last decade, around 300 new compounds have been discovered on a yearly basis from the phylum Porifera [8], and numerous studies have demonstrated a broad range of activities, such as anticancer, anti-inflammatory, immunosuppressive, neurosuppressive, neuroprotective, antiviral, antibacterial and antifungal activities [9]. 

3-Alkyl pyridine alkaloids (3-APAs) are marine natural compounds widely distributed in marine sponges of the order Haplosclerida and are most abundant in the genera *Haliclona*, *Amphimedon* and *Xestospongia* [10,11,12]. These alkaloids contain tetrahydropyridine- or pyridinium moieties connected to aliphatic chains of different lengths, forming monomers [13], dimers, trimers or other more complex polymeric structures [14]**.** Many of these compounds possess significant biological activities such as cytotoxic, antimicrobial, antiviral and anticholinesterase activities [15,16]. Commonly known representatives of the 3-APA family are the cyclostellettamines [17], haliclamines [18], halitoxin [19], manzamines [20], sarains [21] and viscosamine [22]. The search for new bioactive compounds in more harsh environments, like circumpolar regions and deep sea hydrothermal vents, has proven to be successful for filter feeding organisms such as sponges, tunicates and bryozoans [2]. 

Chemical characterization of organisms can be carried out on the level of macromolecules using proteomics [23,24] or by profiling the primary and secondary metabolites (low molecular weight compounds) using metabolomics [25,26]. Metabolomics aims to identify and quantify all low molecular weight metabolites in an organism [27,28]. The simultaneous detection of a wide range of secondary metabolites, known to be species specific, provides an immediate image of the sponge metabolome profile. In the present study, we have used untargeted metabolomics to assay 28 sponge specimens collected at a hydrothermal vent site north of Iceland. Our dataset pointed out a class of compounds with in vitro cytotoxic activity that were isolated and tentatively identified by combining mass spectrometry and ion mobility. 

## 2. Results

**Figure 1:**
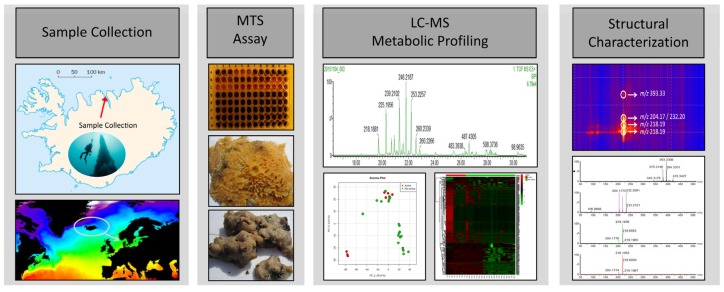
Experimental workflow for the characterization of cytotoxic compounds of sponges collected at the Arnarnesstrýtur vent field.

The aim of this study was to screen extracts from the sponge fauna at the hydrothermal vent site, with the final objective of identifying potential compounds having in vitro cytotoxic activity against a breast cancer cell line. Thus, a MS-based metabolomics approach aligned with the cytotoxicity data was used as a workflow for this project (Figure 1). 

After collection, sponge samples were processed, as described in the experimental section, in order to extract the metabolomes. The 28 sponge extracts were then tested for cytotoxic effects against the SK-BR-3 breast cancer cell line using an in vitro MTS cell proliferation assay. In vitro screening revealed cytotoxic activity in eight specimens against SK-BR-3 breast cancer cells. 

These eight active extracts were obtained from sponges that were identified as *Haliclona rosea*, *Halichondria sitiens*, *Halichondria panicea*, *Myxilla incrustans* and *Lissodendoryx fragilis*. The most active sponge extracts (CH_3_OH/CH_2_Cl_2_) were obtained from the *Haliclona rosea* species, that reduced the viability of the cancer cells by 78%, 69% and 92% at a 33 μg/mL concentration (Table 1 and Figure 1).

### 2.1. Metabolic Profiling

**Figure 2:**
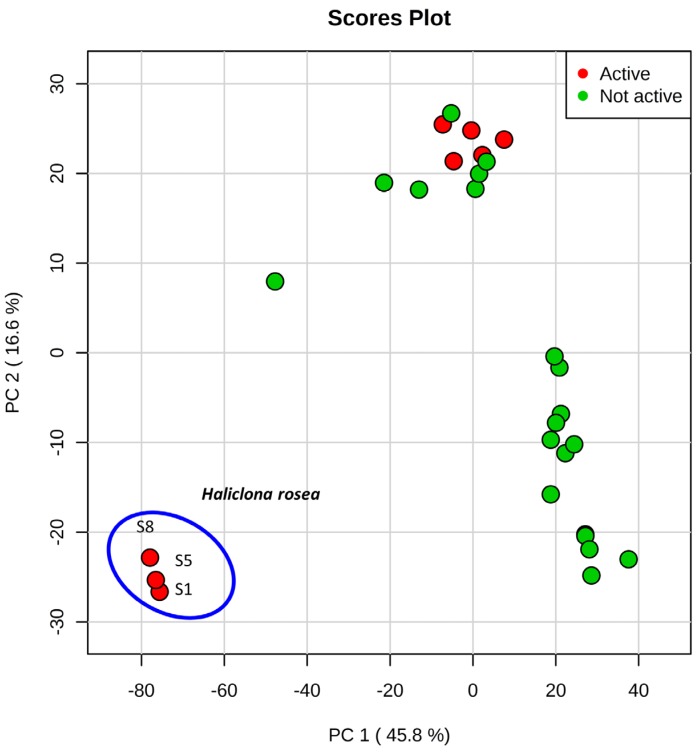
Principal component analysis (PCA) performed on sponge extracts—the clustering of *Haliclona rosea* is shown.

The LC-MS-based untargeted metabolomics approach was used for the initial screening of sponge metabolomes in specimens collected at the hydrothermal vent site. This analysis provided 2107 features, and each of them was characterized by retention time and accurate mass. A better visualization of the sponge metabolomes captured by the LC-MS analysis was obtained by performing principal component analysis (PCA) that was used as a first step for data reduction and prioritization. The principal components were ranked by the variability that they represent in the dataset, with the first principal component accounting for the greatest variability in the data and so on [29]. The first principal component (PC1) accounted for 46% of the total variance and together with the second principal component (PC2) (17% of the total variance), revealed a well-defined cluster formed by the three specimens of *Haliclona rosea*, S1, S5 and S8 (Figure 2a). These three specimens showed in vitro cytotoxicity (70%–90% reduction of viability at 33 μg/mL, Table 1). The PCA clearly shows that the *Haliclona rosea* specimens have different metabolomes (Figure 2). The other five cytotoxic sponge extracts (S2, S3, S4, S6 and S7, Table 1) clustered with the inactive specimens (Figure 2). After comparing the exact masses of the features responsible for the *Haliclona rosea* clustering, with MarinLit (MarinLit database. http://pubs.rsc.org/marinlit/) and Scifinder (Scifinder Database. https://scifinder.cas.org/scifinder), we found a good match with several 3-alkyl pyridine alkaloids, some of which are known to possess cytotoxic activity.

### 2.2. Characterization of 3-APAs in Haliclona rosea Extracts

**Figure 3:**
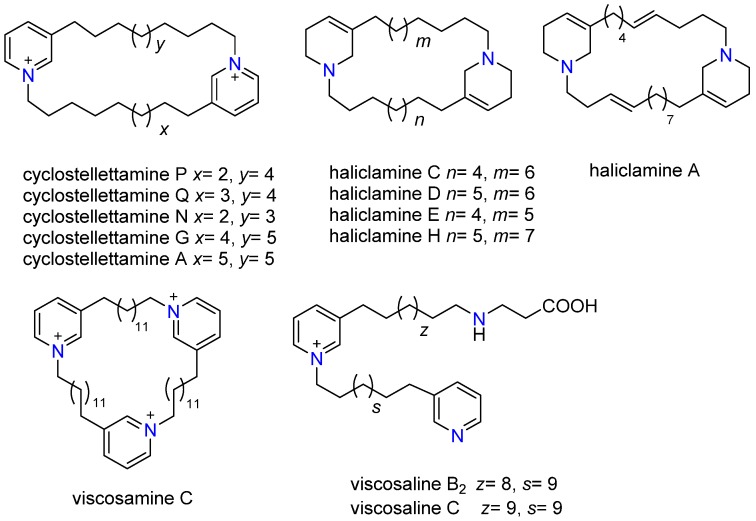
Structures of 3-APA compounds.

**Figure 4:**
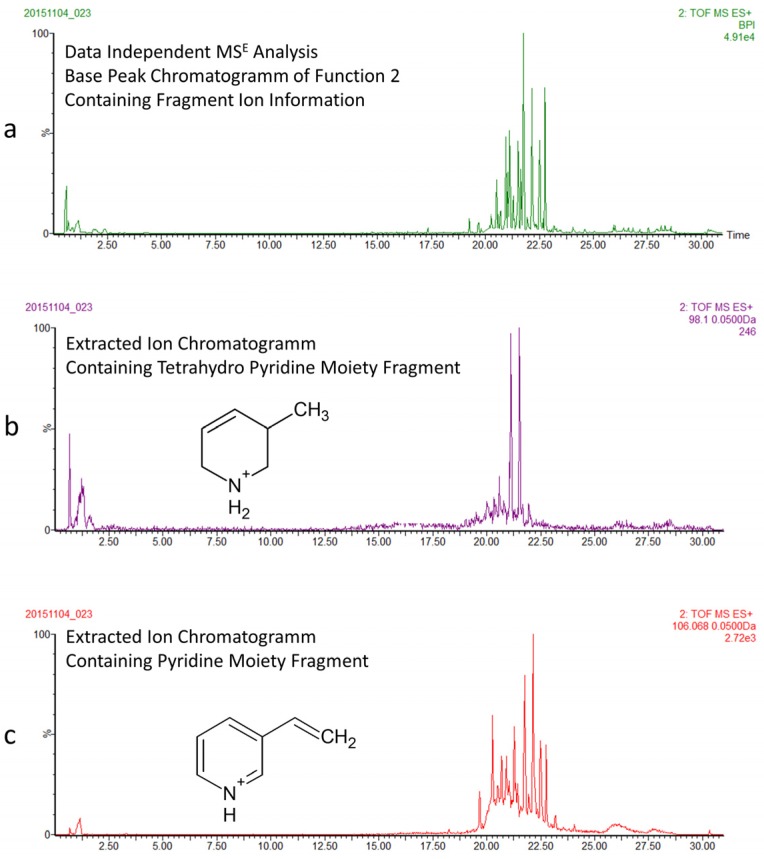
Data independent mass spectrometry (MS^E^) analysis of the butanol extracts of *Haliclona rosea* samples. (**a**) The mass chromatogram at high energy provides fragment ion information of all compounds; (**b**) Extracted ion chromatogram of the diagnostic fragment of tetrahydropyrimidine moiety, representing compounds like the haliclamines; (**c**) Extracted ion chromatogram of the diagnostic fragment of pyridine moiety, representing compounds like the cyclostellettamines, viscosamine and the viscosalines.

Based on the preliminary metabolomics screening results, we focused further experiments on the identification of the 3-APA content in the *Haliclona rosea* specimens (Figure 3). Fractionation was necessary to reduce the chemical complexity of the *Haliclona* metabolome and was performed as described in the experimental section by solvent:solvent partitioning. We investigated the butanol fraction by using another LC-MS method, which combines a longer chromatographic run and data independent mass spectrometry (MS^E^) [30]. The longer run was necessary to separate co-eluting 3-APA compounds, whilst the MS^E^ approach was used because it enables the simultaneous collection of both unfragmented and fragmented ions by generating two discrete and independent interleaved acquisition functions [30,31]. The function 1 (low collision energy) provides unfragmented ions and accurate mass information, while the function 2 (high collision energy) provides fragmented ions. Figure 4a shows the mass chromatogram (function 2 at high energy) of the butanol fraction of *Haliclona rosea* (sample S1). We then used high energy functions and key diagnostic fragments [30] to resolve different 3-APA compounds. Indeed, by extracting the fragment at *m*/*z* 98.0970 (C_6_H_12_N^+^) we were able to differentiate all 3-APA compounds containing the tetrahydropyridine moiety (Figure 4b). The dimeric haliclamine A, C, D, E and H containing the tetrahydropyridine were then tentatively identified by accurate mass and MS/MS information (Table 2). These compounds are recognized by their characteristic doubly charged molecular ions and fragmentation patterns (F_1_ and F_2_) (Table 2 and Figures S1–S5 in Supplementary Materials). The doubly charged molecular ions are: [M + 2H]^2+^ at *m*/*z* 227.2113 for haliclamine A, [M + 2H]^2+^ at *m*/*z* 222.2222 for haliclamine C, [M + 2H]^2+^ at *m*/*z* 229.2259 for haliclamine D, [M + 2H]^2+^ at *m*/*z* 215.2089 for haliclamine E and [M + 2H]^2+^ at *m*/*z* 236.2378 for haliclamine H.

A similar procedure was used to resolve cyclostellettamines, viscosalines and viscosamines that possess the pyridine moiety. In fact, by extracting the fragment at *m*/*z* 106.0657 we were able to resolve these compounds (Figure 4c). Confirmation was achieved by accurate mass and MS/MS information (Table 2 and Figures S6–S9 in Supplementary Materials). The doubly charged molecular ions are: [M + 2H]^2+^ at *m*/*z* 225.1909 for cyclostellettamine Q (C_31_H_51_N_2_), [M + 2H]^2+^ at *m*/*z* 211.1803 for cyclostellettamine N (C_29_H_47_N_2_), [M + 2H]^2+^ at *m*/*z* 239.2110 for cyclostellettamine G (C_33_H_55_N_2_) and [M + 2H]^2+^ at *m*/*z* 246.2150 for cyclostellettamine A (C_34_H_57_N_2_). The trimeric viscosamine C (C_54_H_90_N_3_) was also tentatively identified by using the characteristic triply charged molecular ion, [M + 3H]^3+^ at *m*/*z* 260.2382. The viscosalines produce two doubly charged molecular ions (Table 2 and Figures S11 and S12 in Supplementary Materials). Viscosalines B_2_ and C are compounds containing two pyridine moieties as well as one β-alanine unit and were tentatively identified in these fractions.

Several cyclostellettamine compounds were also found in these extracts (Table 2); cyclostellettamine C, G and N were previously reported in the literature [32,33]. 

### 2.3. Cyclostellettamine P by Ion Mobility Mass Spectrometry

Among the cyclostellettamines tentatively identified (Table 2), we detected cyclostellettamine P (C_30_H_48_N_2_), a new potential analog of the cyclostellettamine family possessing C_9_ and C_11_ alkyl chains, which to the best of our knowledge has never been reported in the literature. Therefore, we tentatively characterized cyclostellettamine P by coupling mass spectrometry with ion mobility working in time-aligned parallel (TAP) fragmentation mode.

The configuration of the SYNAPT system, where collision cells are placed one before and one after the ion mobility (IM) cell, allows an acquisition mode known as time-aligned parallel (TAP) fragmentation [34,35]. During this experiment, it is possible to select a precursor ion of interest and achieve its fragmentation in the first collision cell, before the ion mobility cell. The fragment ions produced can then be separated in the ion mobility cell and subjected to a secondary post-IM fragmentation in the second collision cell. Association of secondary fragment ions to specific drift times of primary fragment ions allows producing a pseudo-MS^3^ experiment [34].

The doubly charged molecular ion of cyclostellettamine P was selected as a precursor ion resulting in five fragments after the pre-IM fragmentation; *m*/*z* 393.33, *m*/*z* 204.17, *m*/*z* 232.20, *m*/*z* 218.19 and *m*/*z* 218.19. The two fragments at *m*/*z* 204.17 and *m*/*z* 232.20 represent the pyridine moieties with the C_9_ and C_11_ chains (Figure S6).

**Figure 5:**
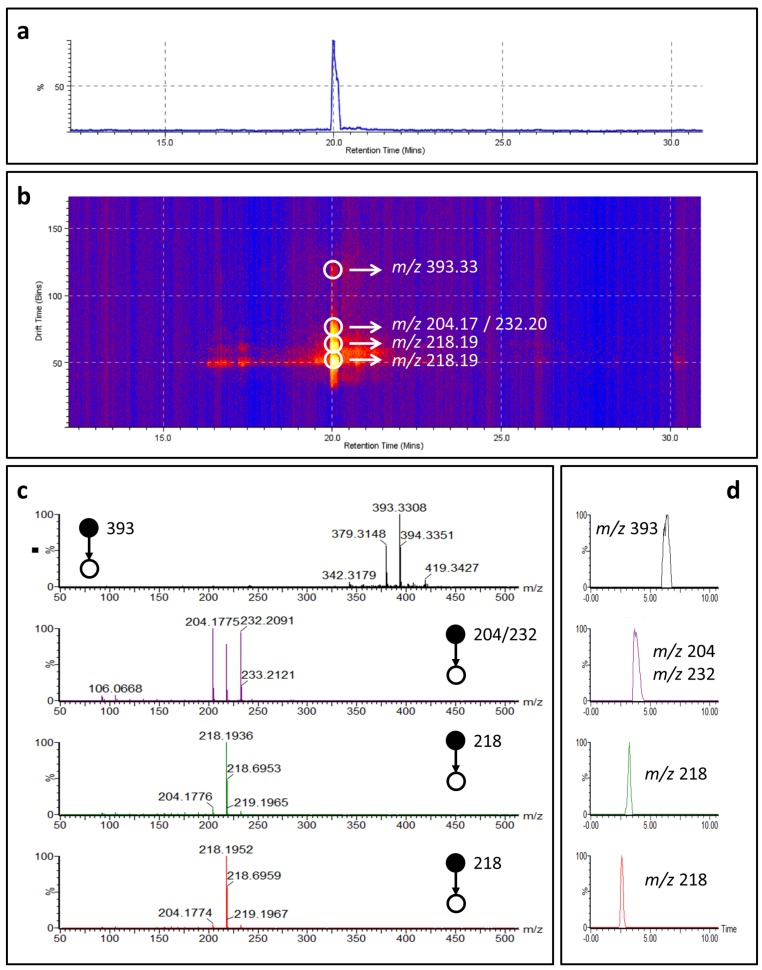
Time-aligned parallel (TAP) fragmentation experiment of cyclostellettamine P. (**a**) Chromatogram of cyclostellettamine P; (**b**) Ion mobility separation of cyclostellettamine P fragment ions (yellow and red dots) plotted as drift time against retention time; (**c**) Mass spectra of cyclostellettamine P fragment ions separated by ion mobility; (**d**) Driftograms of cyclostellettamine P ions.

**Figure 6:**
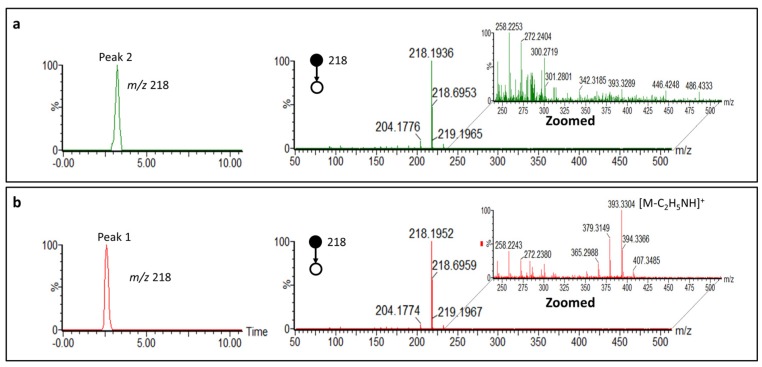
Ion mobility separation of the doubly charged molecular ions of cyclostellettamine P during TAP fragmentation experiment. (**a**) Driftogram and fragmentation spectrum of the isobar at higher drift time; (**b**) Driftogram and fragmentation spectrum of the isobar at lower drift time.

Each fragment ion was separated by ion mobility and dissociated again, providing further information for structural characterization (Figure 5). We were able to separate by ion mobility the two isobar ions at *m*/*z* 218.19 and then associate a specific fragmentation pattern to each of them. These two ions represent the doubly charged cyclostellattamine P (Table 2). We propose that during the ionization process the doubly charged molecular ion generates two different ions at *m*/*z* 218.19 with different mobility. The two fragmentation spectra obtained from each of the two isobars ions at *m*/*z* 218.19 are similar (Figure 6). However, the ion at lower drift time shows specific fragments that suggest the opening of the macrocycle leading to the fragment *m*/*z* 393.33 which results from a loss of *m*/*z* 44.04 [C_2_H_5_NH]^+^ from the singly charged molecular ion (*m*/*z* 437) (Figure 6). The spectrum obtained from the ion at higher mobility suggests a more rigid structure and provides characteristic ions at *m*/*z* 232.20 and *m*/*z* 204.17 obtained via the onium reaction [36].

### 2.4. 3-APAs in Haliclona rosea

**Figure 7:**
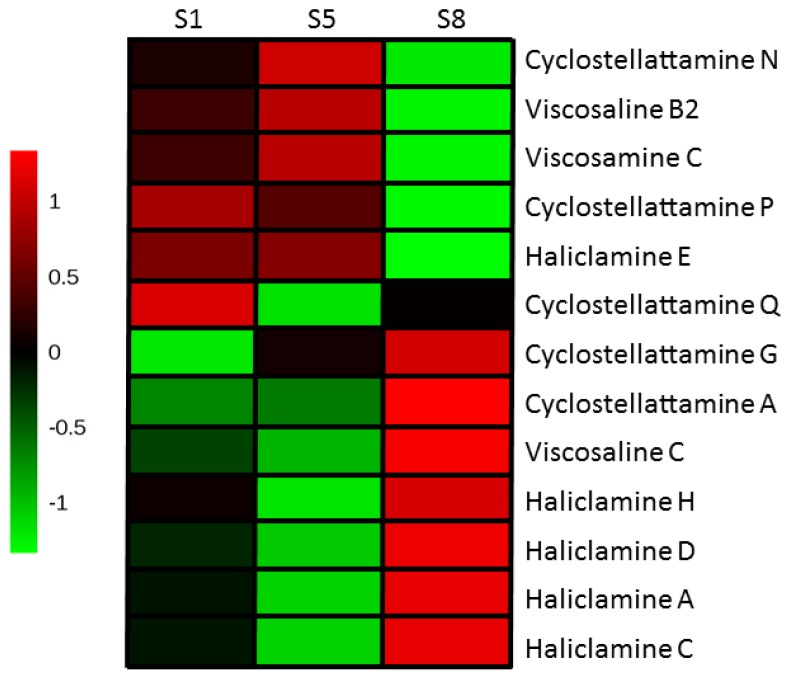
Heat map performed on 3-APA compounds tentatively identified in the three *Haliclona rosea* specimens.

We tentatively identified thirteen different 3-APA compounds in the three *Haliclona rosea* extracts investigated (Table 2 and Figure 7). Two of the *Haliclona rosea* specimens (S1 and S5) contained a similar LC-MS profile for the 3-APA compounds (Figure 7). However, specimen S1 had a higher content of cyclostellettamine Q and of the haliclamines when compared to the same concentration of the S5 specimen (Figure 7). Interestingly, the specimen S8 showed a completely different profile for the 3-APA species. Indeed, this specimen had a higher content of several 3-APA compounds, with the exception of cyclostellettamine N and P, haliclamine E, viscosaline B_2_ and viscosamine C (Figure 7). Thus, a higher composition of the cyclostellettamine P and Q in specimen S1 might be related to the highest cytotoxic activity (Table 1 and Figure 7).

## 3. Discussion

Metabolomics provides a detailed chemical description of complex biological samples. Nevertheless, untargeted metabolomics data are often noisy and a considerable effort is required to tentatively identify unknown compounds. Multivariate statistical analysis is normally used in metabolomics for data reduction and visualization. This study demonstrates the advantage of combining principal component analysis (PCA) and in vitro MTS cell proliferation data in order to prioritize the metabolomics information and group together extracts with higher cytotoxic activity based on their chemical composition (Figure 2). Indeed, the three *Halicona rosea* specimens clustered together due to the presence of the 3-APAs (Table 1, Figure 2), a class of compounds known to possess cytotoxic activities [15,37,38,39]. On the other hand, we were not able to differentiate the other five bioactive sponges that kept clustered within the same group of inactive specimens (Figure 2, Table 1), suggesting that the cytotoxicity of these species is likely due to the presence of compounds with different chemistry. 

Thirteen different 3-APAs compounds were tentatively identified in *Halicona rosea* by using data independent mass spectrometry approaches (MSE) without the need to rerun sample for performing product ion experiments. In addition, we demonstrated the potential of combining ion mobility in metabolomics [40,41] by working in TAP fragmentation mode, which allows to produce pseudo-MS^3^ ions that were used to tentativly characterize and confirm the presence of cyclostellettamine P. Nevertheless, further confirmation is needed by nuclear magnetic resonance (NMR) spectroscopy.

To date, this is the first study focused on the chemical characterization of sponges collected at a shallow water hydrothermal vent site north of Iceland, providing a deeper understanding of the 3-APA composition of *Haliclona rosea* sponges. These sponge specimens were collected during three different periods of the year (March (specimen S8), August (specimen S1) and November (specimen S5)) and each extract exhibited cytotoxicity against the SK-BR-3 breast cancer line. Therefore, a replicated collection over a different timespan would be an interesting approach to study the chemical variation in sponges [42] throughout the year, to optimize the collection of sponges in order to obtain the highest yields of secondary metabolites.

## 4. Materials and Methods

### 4.1. Chemicals

Acetonitrile (CH_3_CN), chloroform (CHCl_3_), dichloromethane (CH_2_Cl_2_), butanol (C_4_H_9_OH), *n*-hexane (C_6_H_14_), formic acid and methanol (CH_3_OH) were of analytical grade and were purchased from Merck (Darmstadt, Germany) and Sigma Aldrich (Seelze, Germany). Water (H_2_O) was obtained using an 18 Ωm Milli-Q system (Millipore, Temecula, CA, USA).

### 4.2. Collection, Identification and Extraction of Sponges

The sponge specimens were collected by SCUBA diving (25 m depth) at the Arnarnesstrýtur hydrothermal vent field in Eyjafjordur N-Iceland (65°51.055′ N–18°11.583′ W). All sponges were identified by using standard morphological methods (gross morphology, spicule size and morphology and organization of the sponge tissue). The identifications were based on [43,44,45]. The samples were kept frozen for 1–2 weeks and lyophilized before extraction. The specimens (S-*sponge sample name*) are also deposited at the Faculty of Pharmaceutical Sciences, University of Iceland. Frozen sponge specimens were lyophilized (30 g dry weight) and extracted three times with 700 mL CH_3_OH/CH_2_Cl_2_ (1:1) using kinetic maceration at room temperature. The extracts were concentrated under reduced pressure at 22 °C using a rotary evaporator to remove the solvent and the residue was re-dissolved in CH_3_OH to be subjected to LC-MS/MS analysis of the extracts.

### 4.3. Partition of Haliclona rosea Extracts and Isolation of Cyclostellettamines

The first step was to subject the extract for a solvent:solvent partition called modified Kupchan partition [46]. The dried extracts were dissolved in 90% aqueous methanol and partitioned against 300 mL of *n*-hexane (*v*/*v*). The water content of the hydro-methanol phase was adjusted to 20% (*v*/*v*) and then to 40% (*v*/*v*) and the solutions were partitioned against CHCl_3_ (500 mL) twice (combined). The hydro-methanol phase was concentrated using a rotary evaporator to remove the methanol and the remaining water extract was partitioned against (150 mL) butanol. The resulting four extracts: hexane fraction (A), chloroform fraction (BC), butanol fraction (D) and water fraction (E) were evaporated to dryness and ready to undergo further chromatographic purifications. The butanol extracts were then used for the data independent mass spectrometry and ion mobility experiments.

### 4.4. MTS Assay—Measurement of Cell Viability (Cytotoxicity)

Effects on cell viability after treatment with marine fractions were measured with a MTS assay on SK-BR-3 breast cancer cell line (obtained from the American Type Culture Collection (ATCC) through LGC Promochem) that overexpresses the HER2/c-erb-2 gene product. SK-BR-3 cells were seeded at a 10^4^ cells per well in 200 μL into 96-well tissue culture plates (Becton Dickinson Labware, Franklin Lakes, NJ, USA). Sponge extracts (CH_3_OH/CH_2_Cl_2_) were dissolved in DMSO and solvent control was added in 33 μg/mL concentration and incubated for 72 h. After incubation for 69 h, 20 μL of the MTS reagent was added per well and incubation continued for a further 3 h (72 h in total) and absorbance at 490 nm was measured (SpectraMax Plus 384 Microplate Reader: Molecular Devices Corporation, Sunnyvale, CA, USA ). The results were expressed as percentage viability compared with solvent treated control cells. Each experiment was carried out in triplicate and repeated twice. Extracts that showed more than 50% decrease in cell viability were selected for secondary screening.

### 4.5. Mass Spectrometry Based Metabolomics

The metabolic profiling of sponge extracts was performed using a Waters ACQUITY UPLC system (Waters, Milford, MA, USA), coupled to a Waters Synapt G1 mass spectrometer equipped with an electrospray ionization (ESI) probe (Waters, Wilmslow, UK). The chromatographic column used was an ACQUITY UPLC BEH C18 (2.1 mm × 100 mm 1.7 μm) (Waters, Milford, MA, USA), which was maintained at 40 °C in a column oven. The gradient system mobile phase consisted of solvent A: H_2_O in 0.1% formic acid and solvent B: CH_3_CN in 0.1% formic acid, at a flow rate of 0.40 mL/min. The injection volume of 6 μL was followed by a linear gradient starting at 85% mobile phase A for 1.0 min up to 100% of mobile phase B in 14.0 min. The gradient was held for 4.0 min before returning to the initial conditions at 18.5 min and then held for another 2.1 min. The total chromatographic run time was 21 min. The sample manager temperature was maintained at 20.0 °C. The mass spectrometer was optimized for analyzing the extracts using LC-MS/MS method. The ionization source parameters were: capillary voltage 3.5 kV; cone voltage 15 V; source temperature 120 °C; desolvation temperature 450 °C at a flow rate of 700 L·h^−1^ (N_2_); cone gas flow rate 50 L·h^−1^. Data acquisition was carried out using MassLynx 4.1 software (Waters, Wilmslow, UK).

### 4.6. Data Processing and Analysis

Progenesis QI (Nonlinear Dynamics, Newcastle, UK) was used for processing metabolomics data. Isotope and adduct removal was applied to reduce the number of features detected. Marinlit was used as the main database to search for known compounds previously identified in sponges.

Principal component analysis (PCA) was performed by using MetaboAnalyst [47]. Before PCA, data was normalized by the sum, log transformed and then scaled by using unite variance.

### 4.7. Ion Mobility Mass Spectrometry

An UPLC system (ACQUITY UPLC Waters, Milford, MA, USA) was coupled in line with a QTOF mass spectrometer (Synapt G2, Waters, Wilmslow, UK) operating in positive mode. Data was acquired in data independent mass spectrometry mode (MS^E^) from *m*/*z* 50 to 1200 generating two discrete and independent intervaled acquisition functions. Argon served as collision gas and in the collision energy in the trap cell was 4 eV (Function 1, low energy); in the transfer cell, it ramped from 30 to 40 eV (Function 2, high energy). UPLC separation was performed using a BEH C18 1.7 μm (2.1 × 100 mm) column. The capillary and cone voltage were 1.5 and 30 V, respectively. The source and desolvation temperature were 120 and 500 °C and the desolvation gas flow was 800 L/h. Leucine enkephalin (2 ng/μL) was used as lock mass (*m*/*z* 556.2771). Ion mobility experiments were performed using nitrogen as ion mobility gas, which flowed at a rate of 90 mL/min (3.2 mbar), with a wave velocity of 600 m/s and a wave height of 40 V. The EDC delay coefficient was specified as 1.58 V. Data was acquired in time-aligned parallel (TAP) fragmentation mode [34] from *m*/*z* 50 to 1000, generating MS^3^ fragments. Argon served as collision gas and the collision energy in the trap cell and in the transfer cell ranged from 20 to 30 eV.

## Figures and Tables

**Table 1:** Cytotoxic sponges collected at the hydrothermal vent site (Arnarnesstrýtur). Sponge extracts were tested in a 33 μg/mL concentration in DMSO.

Sample Name	MTS Results (% Viability of Cells)	Identification	Depth (m)
S1	8%	*Haliclona rosea*	24
S2	29%	*Halichondria sitiens*	24
S3	51%	*Myxilla incrustans*	24
S4	18%	*Halichondria panicea*	27
S5	31%	*Haliclona rosea*	25
S6	31%	*Lissodendoryx fragilis*	24
S7	13%	*Halichondria panicea*	27
S8	22%	*Haliclona rosea*	28

**Table 2:** 3-APA compounds tentatively identified in the *Haliclona rosea* extracts.

Pyr/THP (Moiety)	Oligomer	Compound Name	Formula	HRMS [M + H]^+^ (Calcd. Mass)	HRMS [M + 2H]^2+^ (Calcd. Mass)	MS/MS Main Fragments (F_1_ and F_2_)
Pyr	dimer (cyclic)	**Cyclostellettamine P**	C_30_H_49_N_2_	437.3896 Δ*ppm* 1	218.1909	204.1752 (C_14_H_22_N)
						232.2065 (C_16_H_26_N)
Pyr	dimer (cyclic)	**Cyclostellettamine Q **	C_31_H_51_N_2_	451.4052 Δ*ppm* 3	225.1909	218.1909 (C_15_H_24_N)
						232.2065 (C_16_H_26_N)
Pyr	dimer (cyclic)	**Cyclostellettamine N**	C_29_H_47_N_2_	423.3779 Δ*ppm* 9	211.1803	204.1752 (C_14_H_22_N)
						218.1909 (C_15_H_24_N)
Pyr	dimer (cyclic)	**Cyclostellettamine G**	C_33_H_55_N_2_	479.4365 Δ*ppm* 10	239.211	232.2065 (C_16_H_26_N)
						246.1752 (C_17_H_28_N)
Pyr	dimer (cyclic)	**Cyclostellettamine A**	C_34_H_57_N_2_	493.4522 Δ*ppm* 3	246.215	261.2331 (C_17_H_29_N)
						261.2331 (C_17_H_29_N)
THP	dimer (cyclic)	**Haliclamine A**	C_31_H_53_N_2_	453.4209 Δ*ppm* 8	227.2113	204.1752 (C_14_H_22_N)
						246.2222 (C_17_H_28_N)
THP	dimer (cyclic)	**Haliclamine C**	C_30_H_55_N_2_	443.4365 Δ*ppm* 8	222.2222	208.2065 (C_14_H_26_N)
						236.2378 (C_16_H_30_N)
THP	dimer (cyclic)	**Haliclamine D**	C_31_H_57_N_2_	457.4522 Δ*ppm* 6	229.2259	222.2222 (C_15_H_28_N)
						236.2378 (C_16_H_30_N)
THP	dimer (cyclic)	**Haliclamine E**	C_29_H_53_N_2_	429.4209 Δ*ppm* 1	215.2089	208.2065 (C_14_H_26_N)
						222.2222 (C_15_H_28_N)
THP	dimer (cyclic)	**Haliclamine H**	C_32_H_59_N_2_	471.4678 Δpp*m* 4	236.2378	222.2222 (C_15_H_28_N)
						250.2535 (C_17_H_32_N)
Pyr	trimer (cyclic)	**Viscosamine C**	C_54_H_90_N_3_	260.2382 [M]^3+^	n/a	390.8637 (C_54_H_91_N_3_)
						389.8637 (C_54_H_89_N_3_)
Pyr	Linear	**Viscosaline B_2_**	C_38_H_64_N_3_O_2_	594.4999 Δ*ppm* 4	246.2191/297.7537	253.2321 (C_35_H_58_N_2_)
						267.7429 (C_36_H_61_N_3_)
Pyr	Linear	**Viscosaline C**	C_39_H_67_N_3_O_2_	608.5155 Δpp*m* 5	253.2264/ 304.7621	260.2376 (C_36_H_60_N_2_)
						274.7509 (C_37_H_63_N_3_)

## Supplementary Materials

The following are available online at www.mdpi.com/1660-3997/15/2/52/s1: Figure S1: Mass fragmentation of haliclamine A induced by ESIMS (positive mode) on Waters Synapt (QTOF), Figure S2: Mass fragmentation of haliclamine C induced by ESIMS (positive mode) on Waters Synapt (QTOF), Figure S3: Mass fragmentation of haliclamine D induced by ESIMS (positive mode) on Waters Synapt (QTOF), Figure S4: Mass fragmentation of haliclamine E induced by ESIMS (positive mode) on Waters Synapt (QTOF), Figure S5: Mass fragmentation of haliclamine H induced by ESIMS (positive mode) on Waters Synapt (QTOF), Figure S6: Mass fragmentation of cyclostellettamine P induced by ESIMS (positive mode) on Waters Synapt (QTOF), Figure S7: Mass fragmentation of cyclostellettamine Q induced by ESIMS (positive mode) on Waters Synapt (QTOF), Figure S8: Mass fragmentation of cyclostellettamine N induced by ESIMS (positive mode) on Waters Synapt (QTOF), Figure S9: Mass fragmentation of cyclostellettamine G induced by ESIMS (positive mode) on Waters Synapt (QTOF), Figure S10: Mass fragmentation of viscosamine C induced by ESIMS (positive mode) on Waters Synapt (QTOF), Figure S11: Mass fragmentation of viscosaline B2 induced by ESIMS (positive mode) on Waters Synapt (QTOF), Figure S12: Mass fragmentation of viscosaline C induced by ESIMS (positive mode) on Waters Synapt (QTOF).
