# Supplementary material for: Metabolic Profiling as a Screening Tool for Cytotoxic Compounds: Identification of 3-Alkyl Pyridine Alkaloids from Sponges Collected at a Shallow Water Hydrothermal Vent Site North of Iceland
Source: Mar Drugs. 2017 Feb 22;15(2):52. doi: 10.3390/md15020052 (PMC5334632; doi:10.3390/md15020052)
Supplement: Supplementary file 1 [file marinedrugs-15-00052-s001.pdf]

Supplementary Materials: Metabolic Profiling as a Screening Tool for Cytotoxic Compounds: Identification of 3-Alkyl Pyridine Alkaloids from Sponges Collected at a Shallow Water Hydrothermal Vent Site North of Iceland

Eydis Einarsdottir, Manuela Magnusdottir, Giuseppe Astarita, Matthias Köck, Helga M. Ögmundsdottir, Margret Thorsteinsdottir, Hans Tore Rapp, Sesselja Omarsdottir, and Giuseppe Paglia

Figure S1. Mass fragmentation of haliclamine A induced by ESIMS (positive mode) on Waters Synapt (QTOF).

Figure S2. Mass fragmentation of haliclamine C induced by ESIMS (positive mode) on Waters Synapt (QTOF).

Figure S3. Mass fragmentation of haliclamine D induced by ESIMS (positive mode) on Waters Synapt (QTOF).

Figure S4. Mass fragmentation of haliclamine E induced by ESIMS (positive mode) on Waters Synapt (QTOF).

Figure S5. Mass fragmentation of haliclamine H induced by ESIMS (positive mode) on Waters Synapt (QTOF).

Figure S6. Mass fragmentation of cyclostellettamine P induced by ESIMS (positive mode) on Waters Synapt (QTOF).

Figure S7. Mass fragmentation of cyclostellettamine Q induced by ESIMS (positive mode) on Waters Synapt (QTOF).

Figure S8. Mass fragmentation of cyclostellettamine N induced by ESIMS (positive mode) on Waters Synapt (QTOF).

Figure S9. Mass fragmentation of cyclostellettamine G induced by ESIMS (positive mode) on Waters Synapt (QTOF).

Figure S10. Mass fragmentation of viscosamine C induced by ESIMS (positive mode) on Waters Synapt (QTOF).

Figure S11. Mass fragmentation of viscosaline B2 induced by ESIMS (positive mode) on Waters Synapt (QTOF).

Figure S12. Mass fragmentation of viscosaline C induced by ESIMS (positive mode) on Waters Synapt (QTOF).

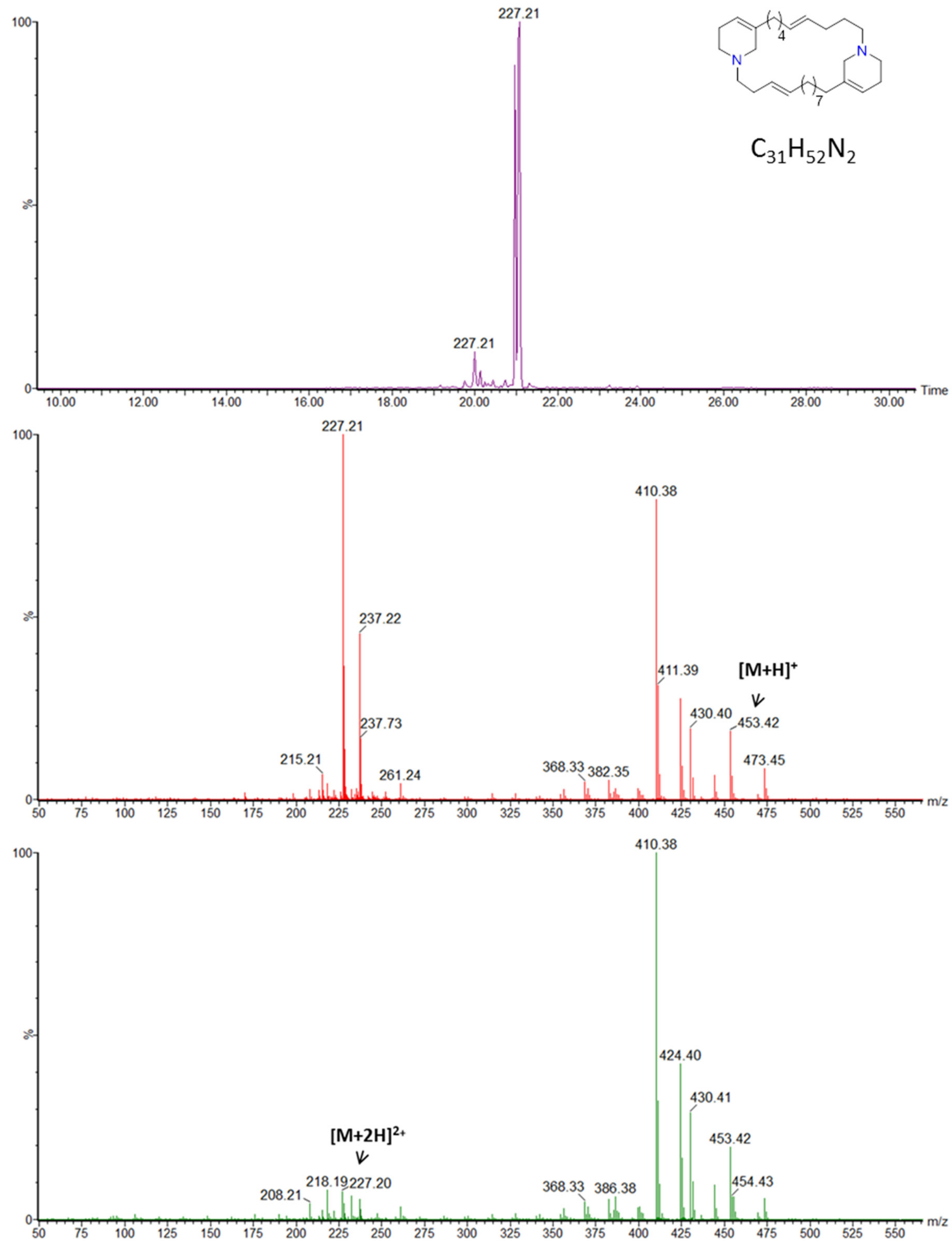

Figure S1. Mass fragmentation of haliclamine A induced by ESIMS (positive mode) on Waters Synapt (QTOF).

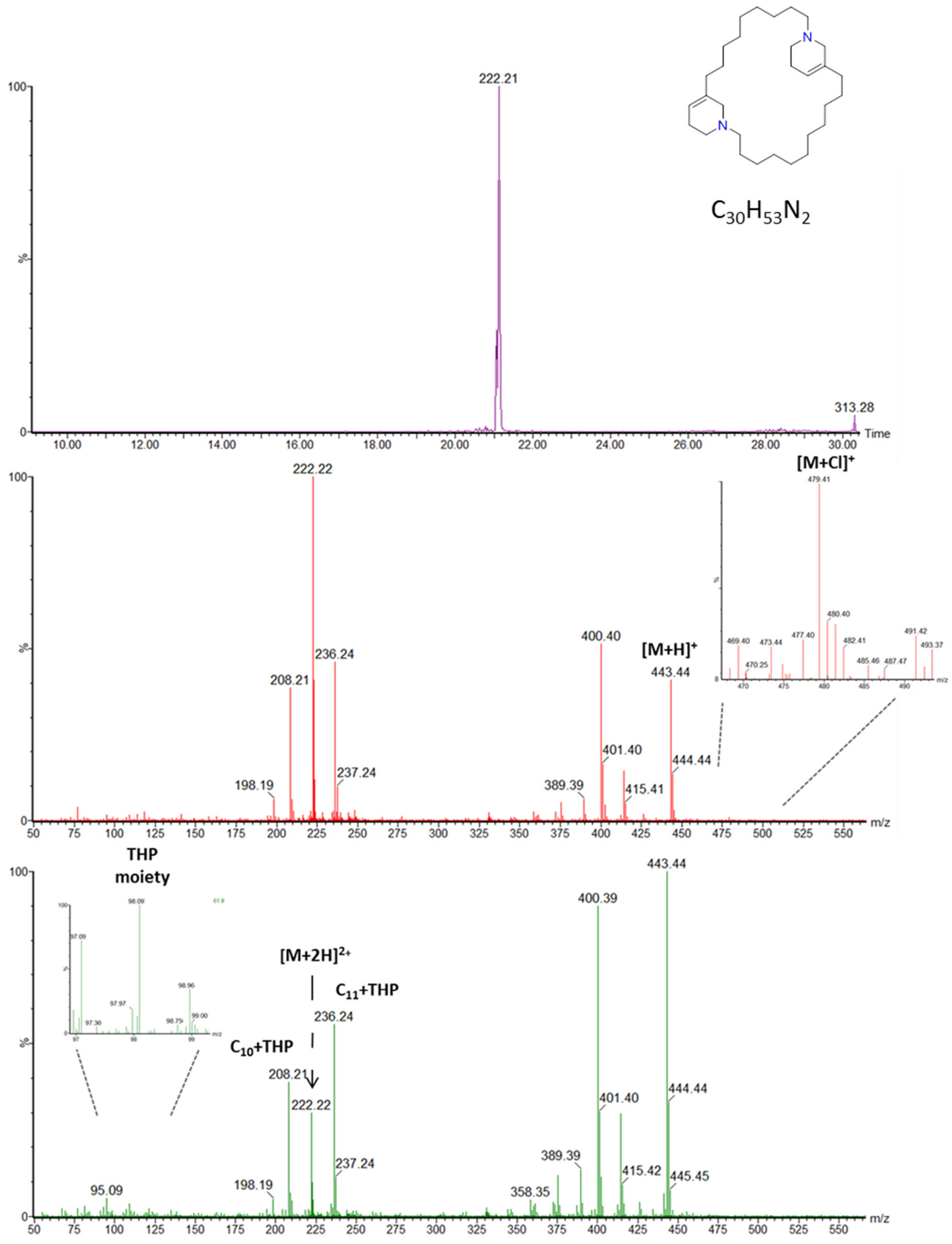

Figure S2. Mass fragmentation of haliclamine C induced by ESIMS (positive mode) on Waters Synapt (QTOF).

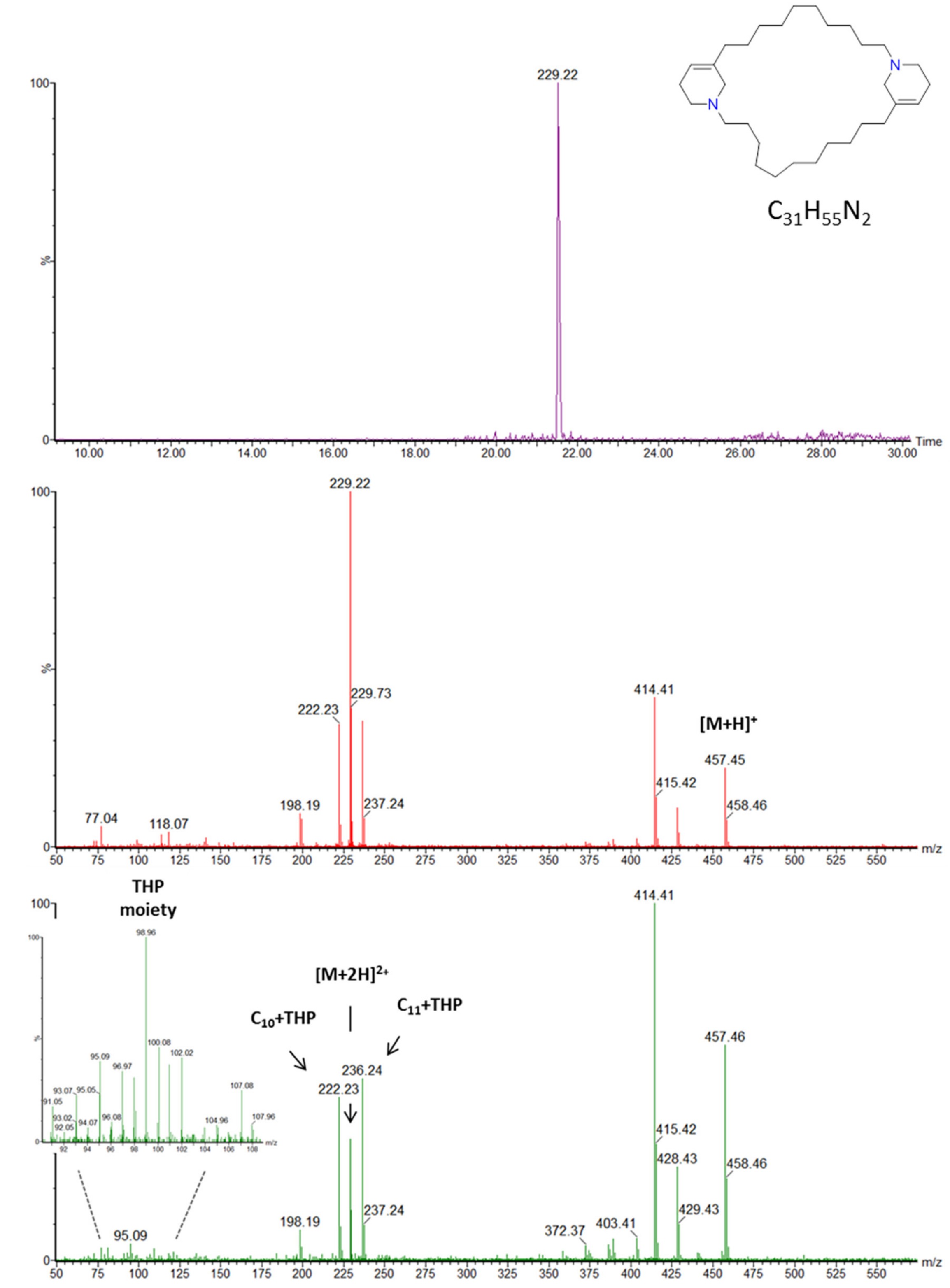

Figure S3. Mass fragmentation of haliclamine D induced by ESIMS (positive mode) on Waters Synapt (QTOF).

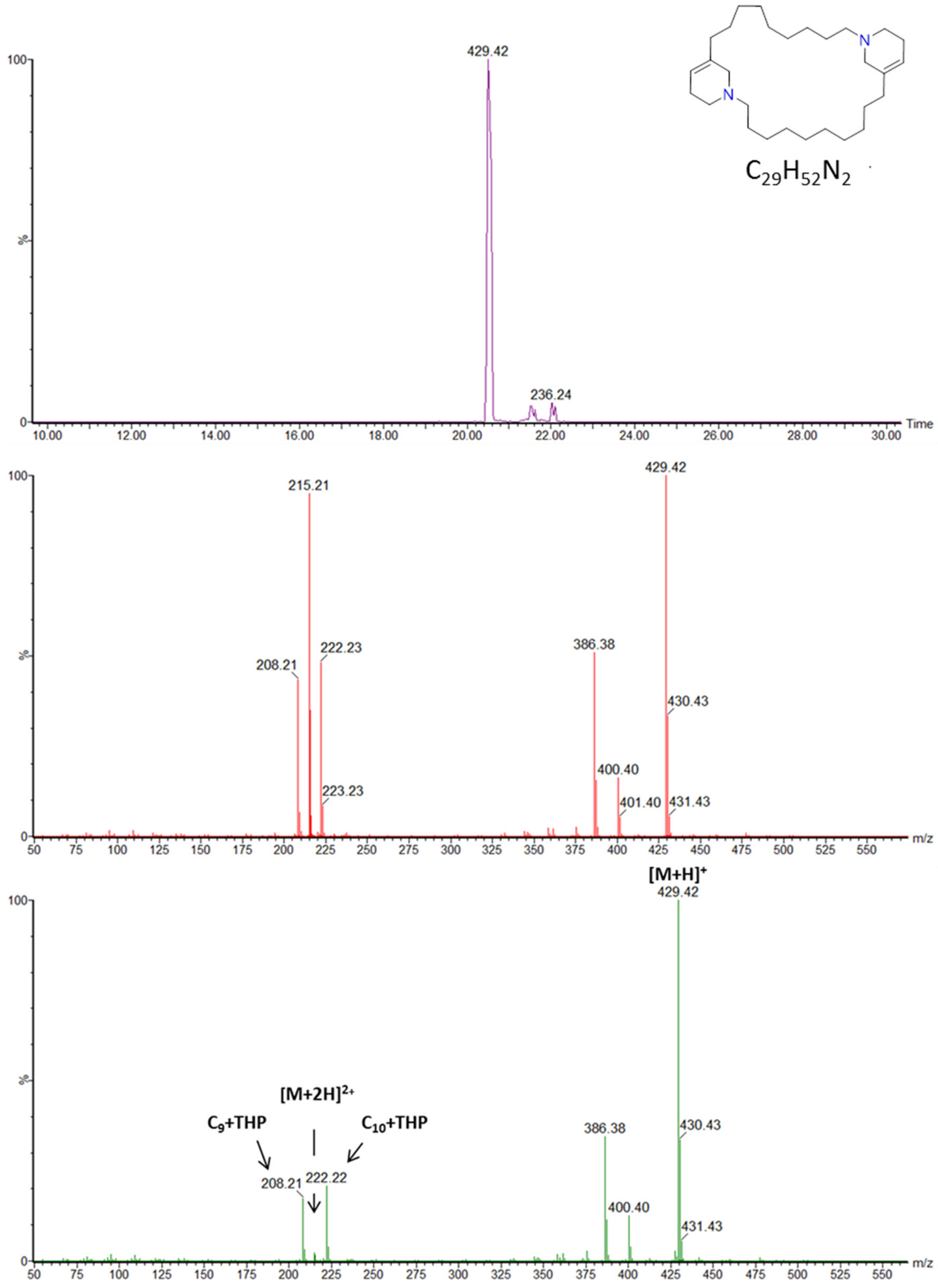

Figure S4. Mass fragmentation of haliclamine E induced by ESIMS (positive mode) on Waters Synapt (QTOF).

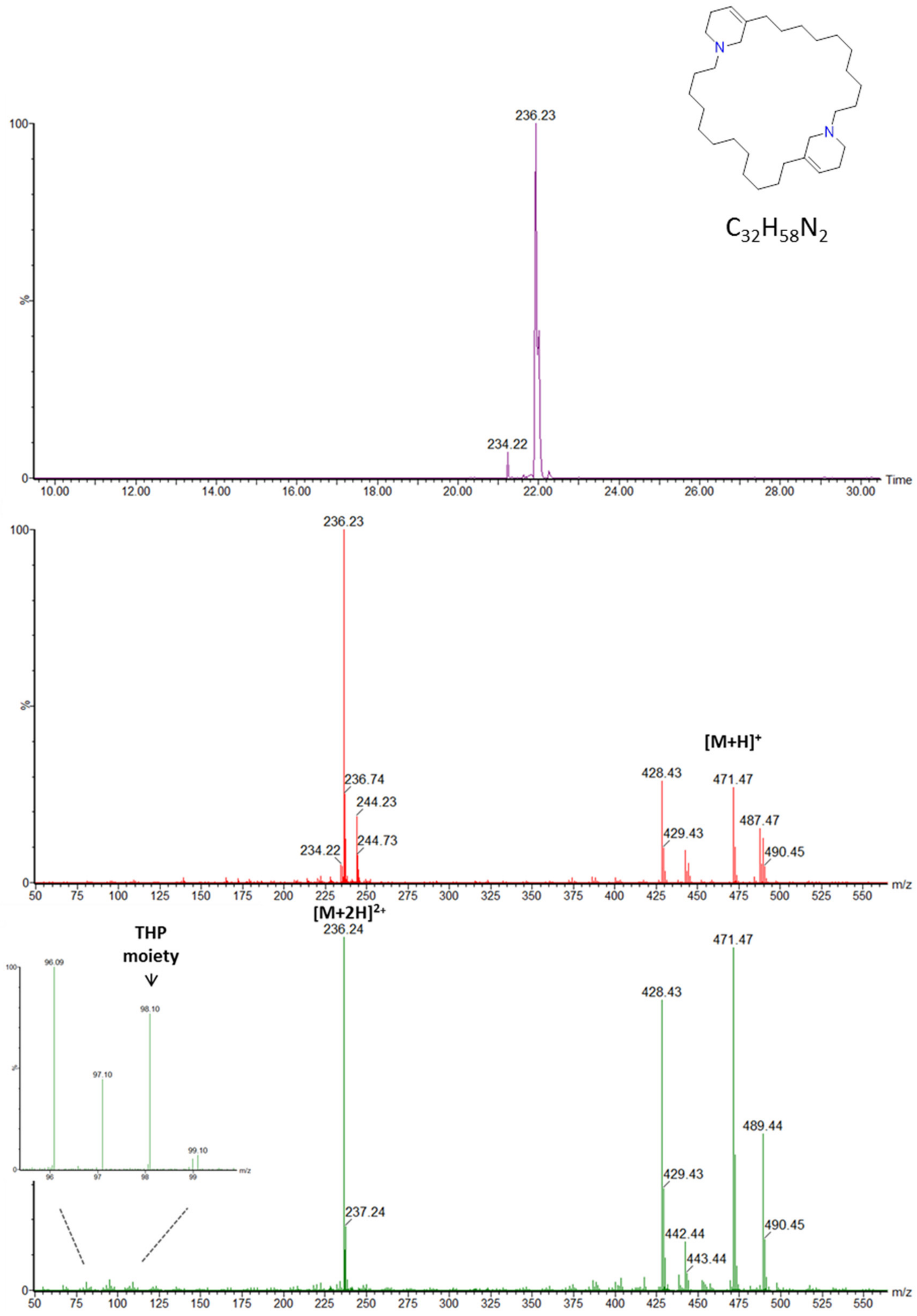

Figure S5. Mass fragmentation of haliclamine H induced by ESIMS (positive mode) on Waters Synapt (QTOF).

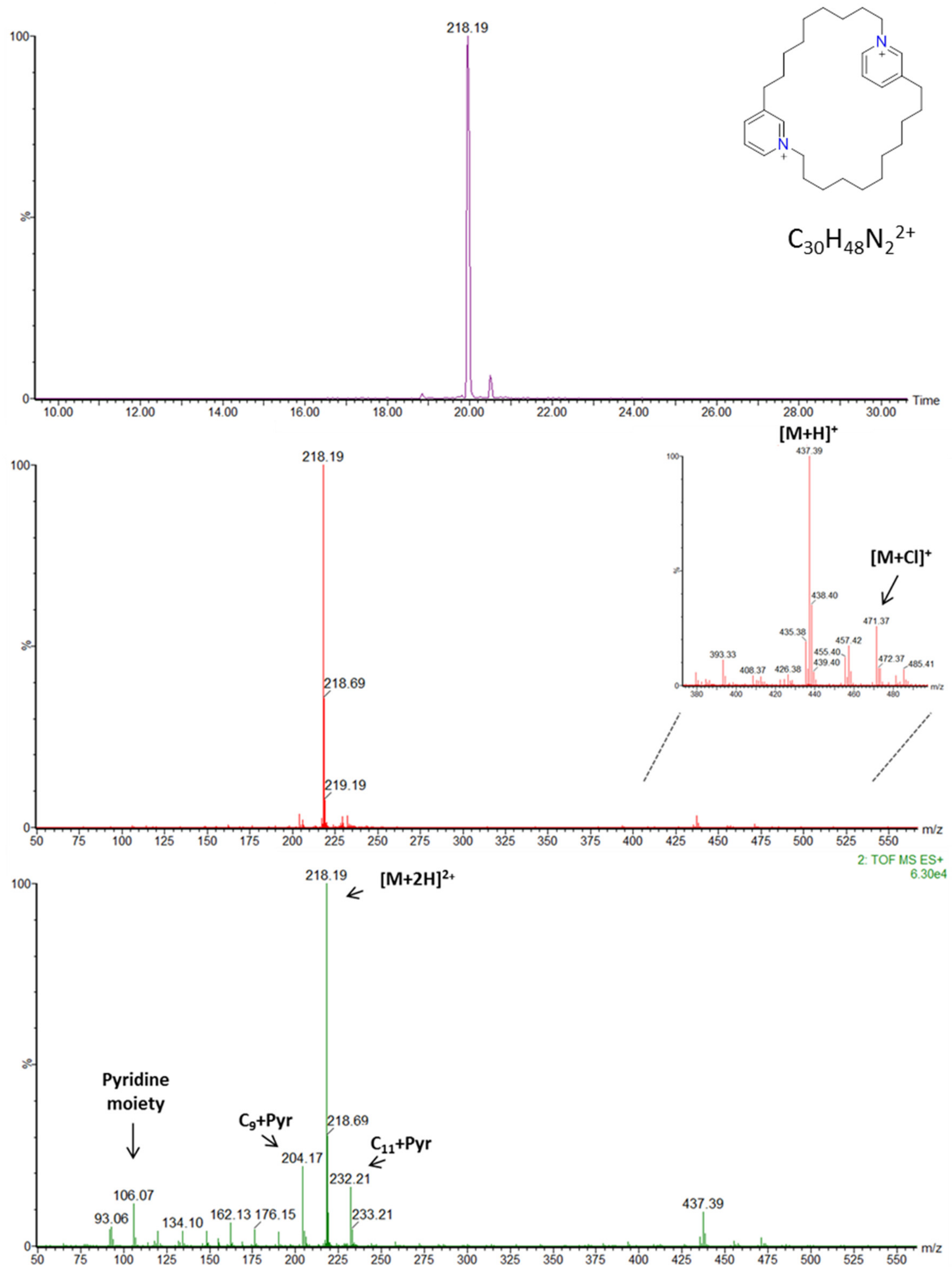

Figure S6. Mass fragmentation of cyclostellettamine P induced by ESIMS (positive mode) on Waters Synapt (QTOF).

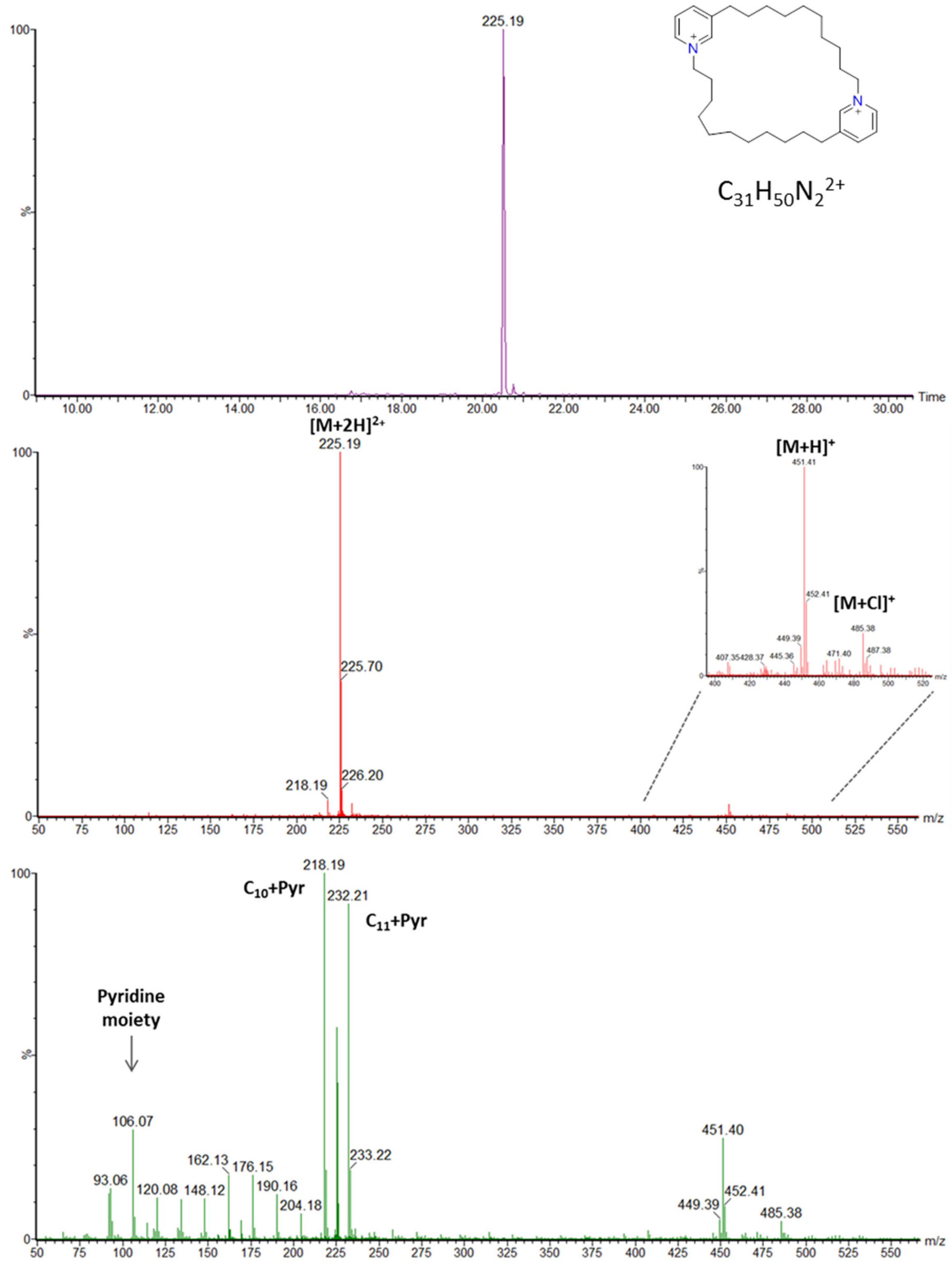

Figure S7. Mass fragmentation of cyclostellettamine Q induced by ESIMS (positive mode) on Waters Synapt (QTOF).

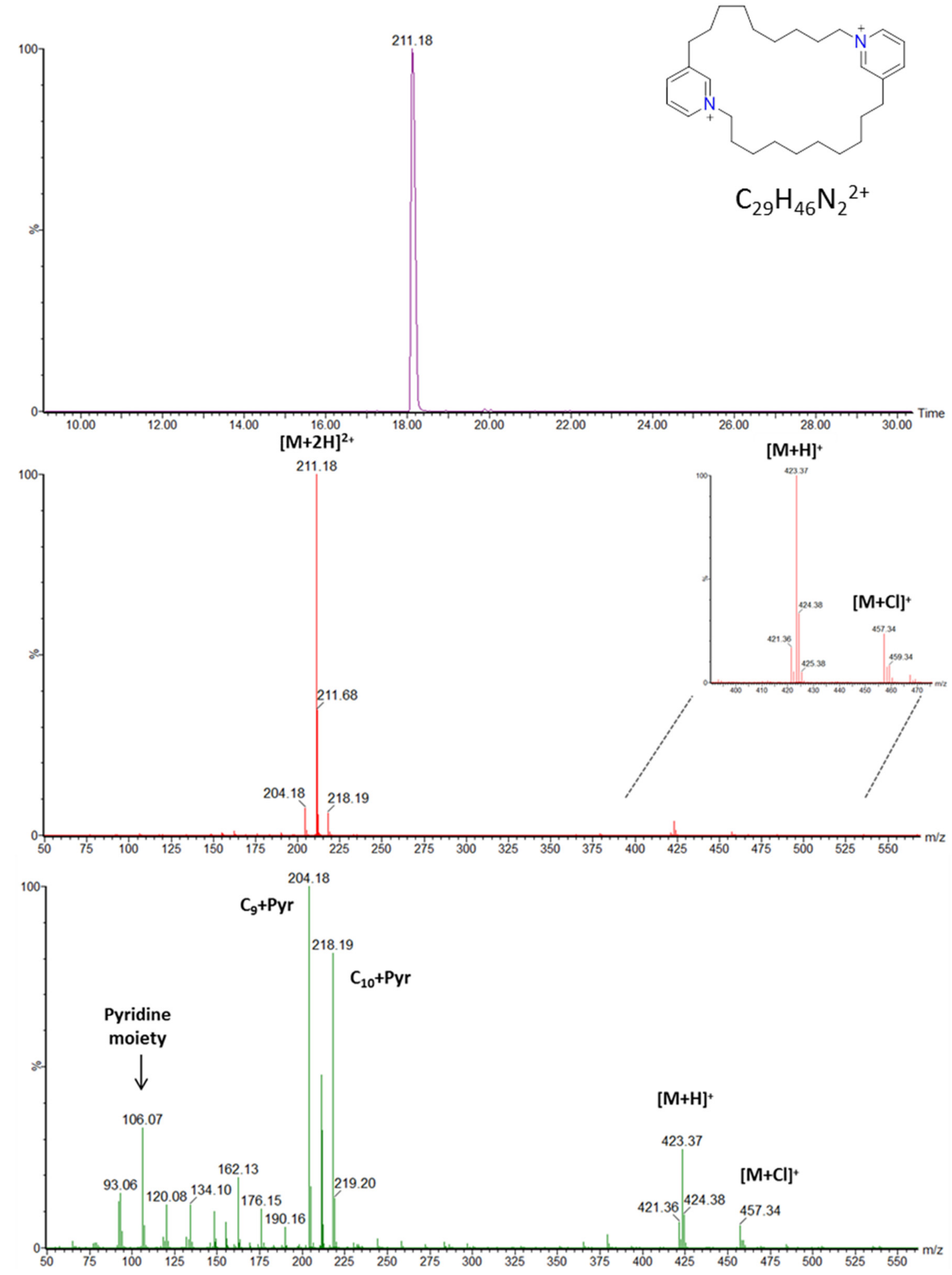

Figure S8. Mass fragmentation of cyclostellettamine N induced by ESIMS (positive mode) on Waters Synapt (QTOF).

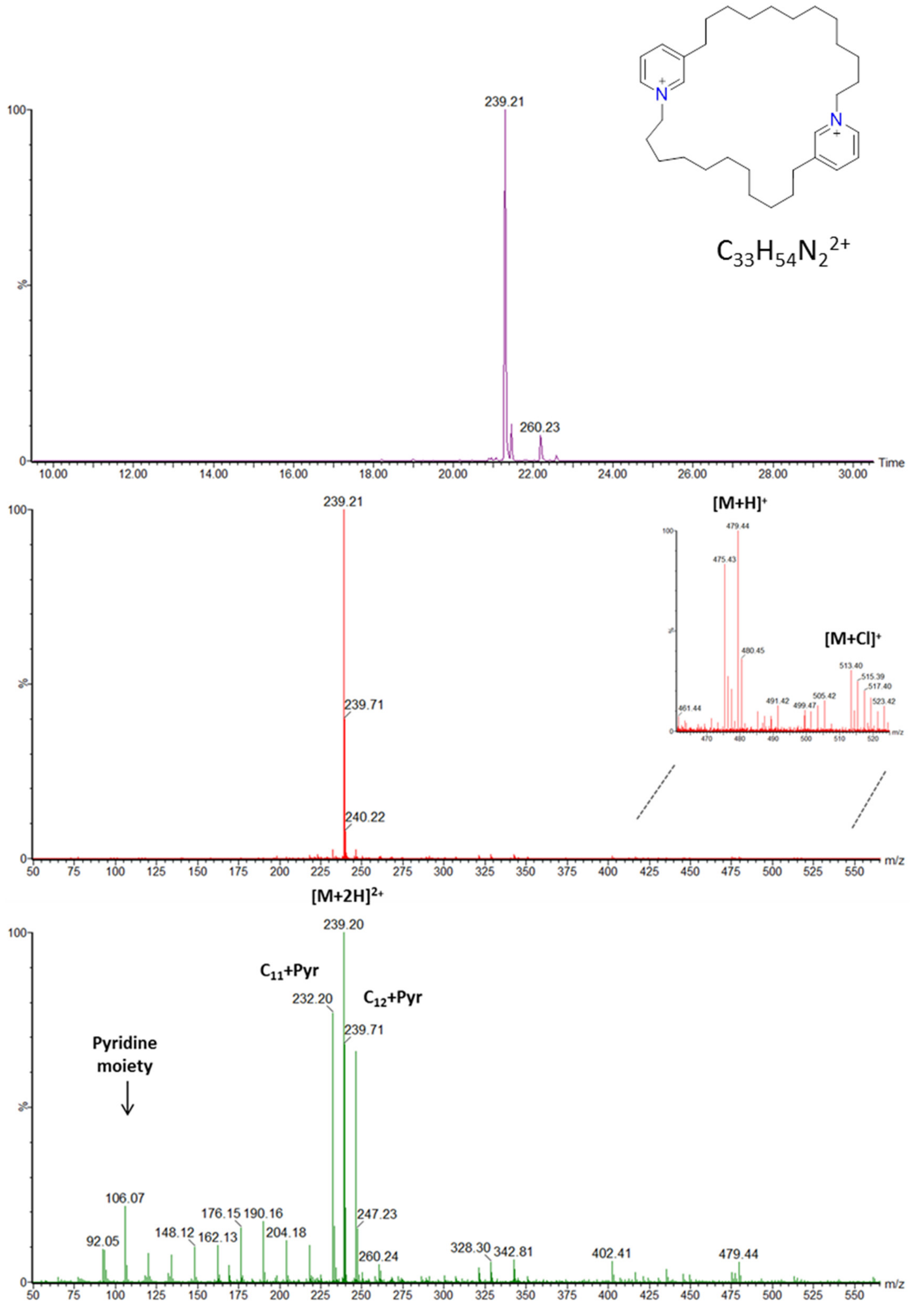

Figure S9. Mass fragmentation of cyclostellettamine G induced by ESIMS (positive mode) on Waters Synapt (QTOF).

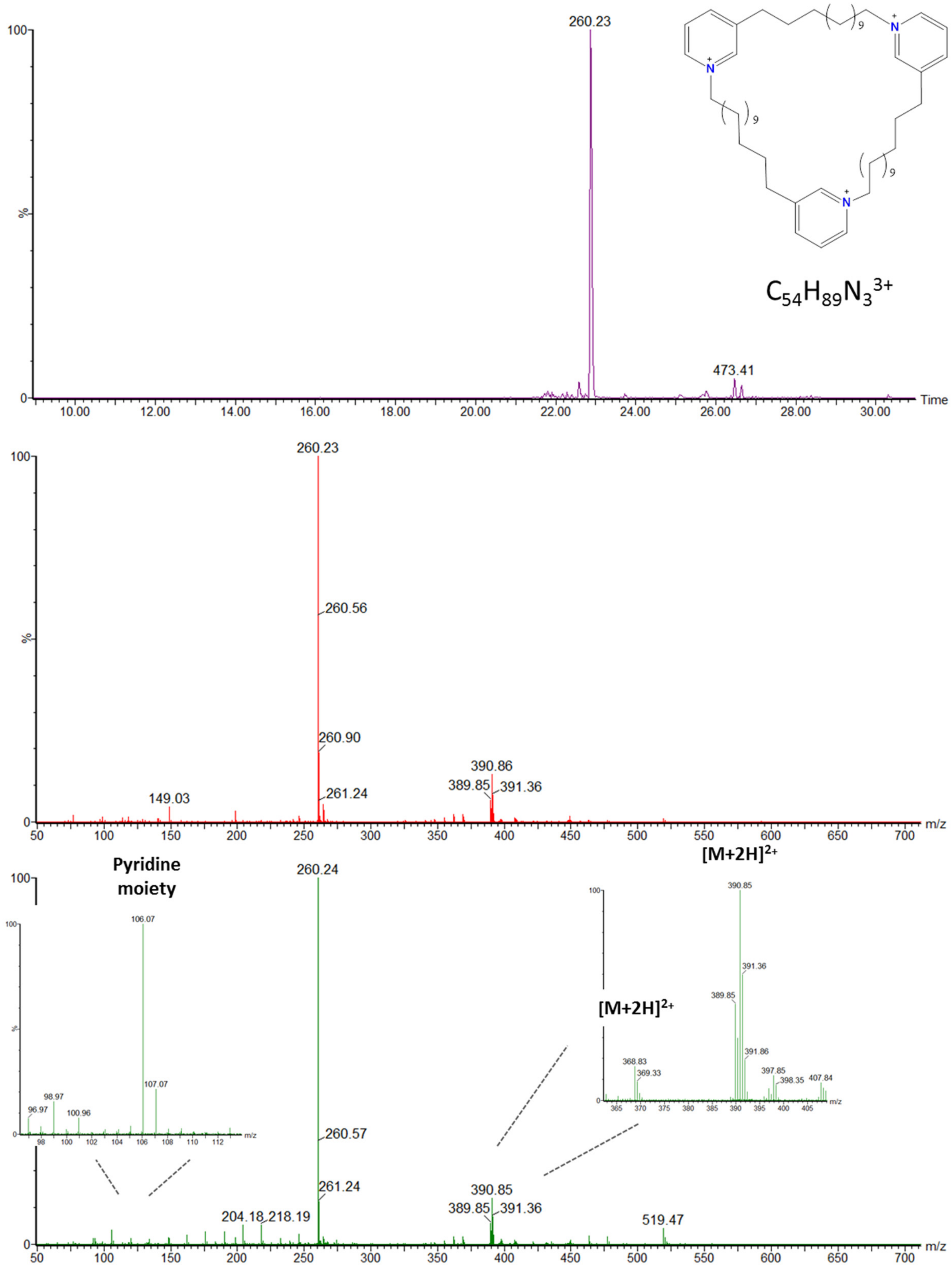

Figure S10. Mass fragmentation of viscosamine C induced by ESIMS (positive mode) on Waters Synapt (QTOF).

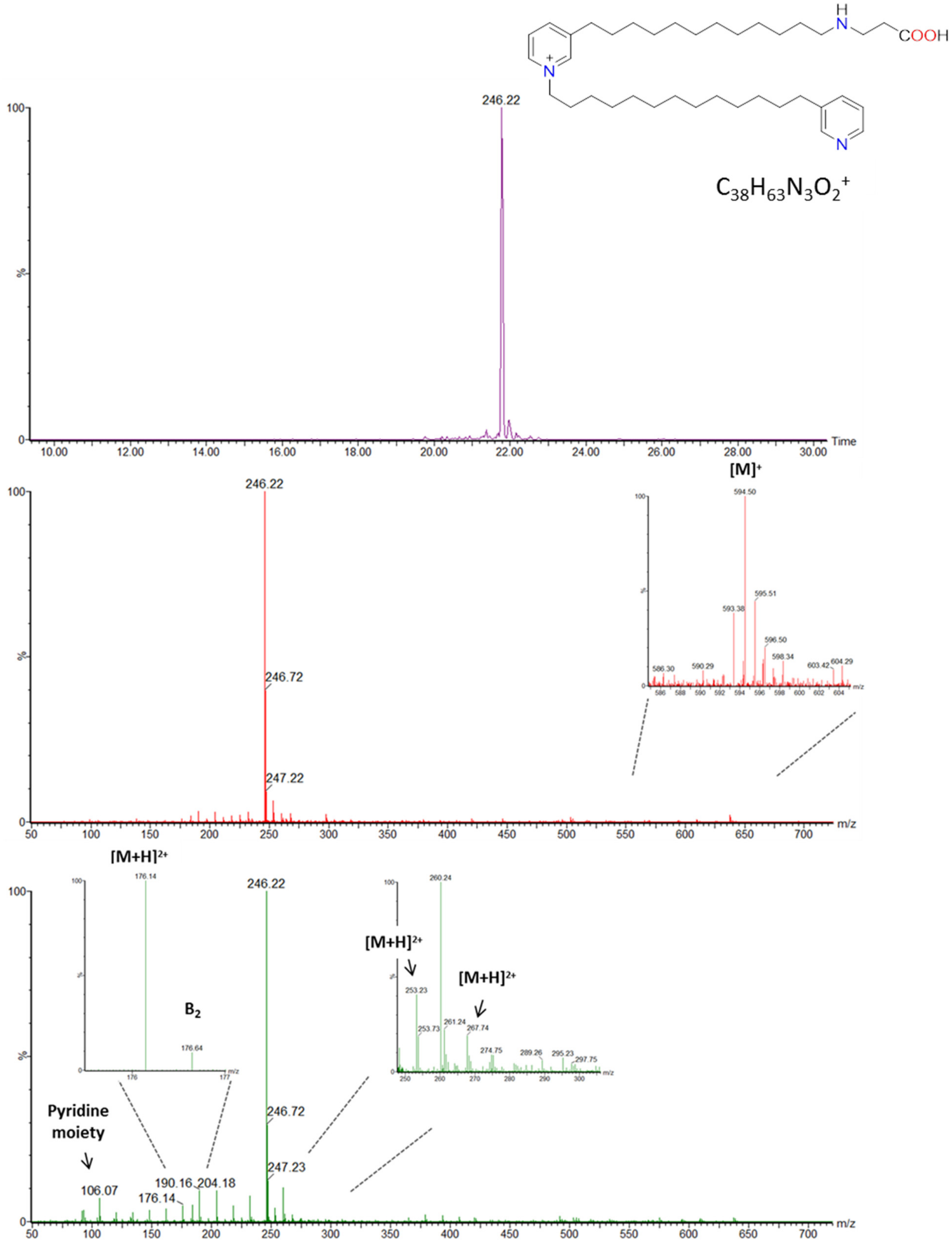

Figure S11. Mass fragmentation of viscosaline B2 induced by ESIMS (positive mode) on Waters Synapt (QTOF).

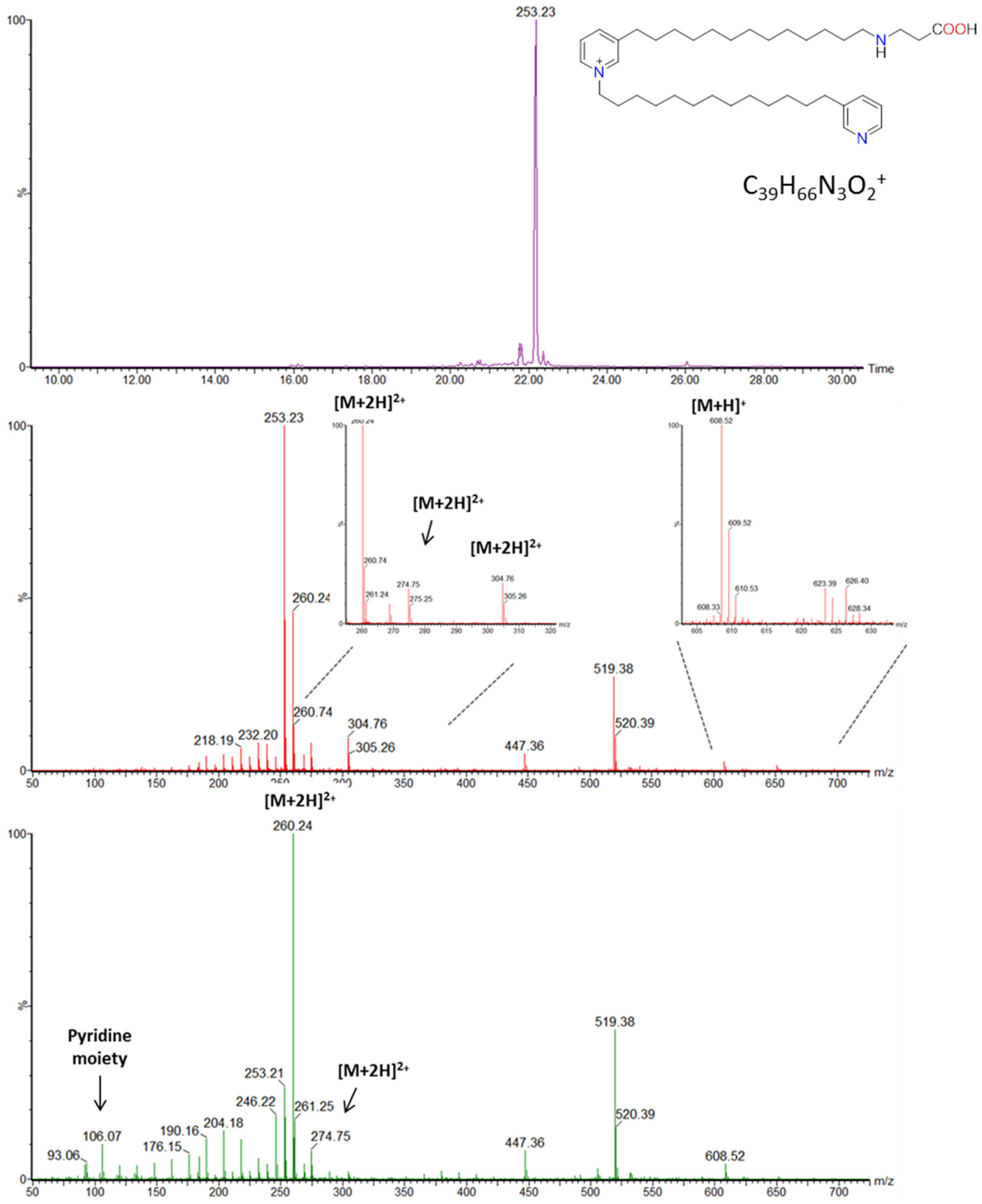

Figure S12. Mass fragmentation of viscosaline C induced by ESIMS (positive mode) on Waters Synapt (QTOF).
